# Hybrid Cardiac Telerehabilitation After Acute Coronary Syndrome: Self-selection Predictors and Outcomes

**DOI:** 10.5195/ijt.2023.6475

**Published:** 2023-05-11

**Authors:** José Bernardo Ferreira, Margarida Cabral, Rita Santos, Marta Ferreira, Rui Fonseca-Pinto, Alexandre Antunes, Filipa Januário

**Affiliations:** 1 Physical and Rehabilitation Medicine Department, Leiria Hospital Centre, Leiria, Portugal; 2 Cardiology Department, Leiria Hospital Centre, Leiria, Portugal; 3 Centro De Matemática, Universidade Do Minho, Braga, Portugal; 4 CiTechcare-Center for Innovative Care and Health Technology, Polytechnic of Leiria, Leiria, Portugal

**Keywords:** Acute coronary syndrome, Cardiac rehabilitation, Telerehabilitation

## Abstract

**Aims:**

To evaluate the effectiveness of a hybrid cardiac telerehabilitation (HCTR) program after acute coronary syndrome (ACS) on patient quality of life (QoL) and physical activity indices throughout phases 2-3 and establish predictors for hybrid program self-selection.

**Methodology:**

This single-centre longitudinal retrospective study included patients who attended a cardiac rehabilitation program (CRP) between 2018-2021. Patients self-selected between two groups: Group 1 – conventional CRP (CCRP); Group 2 – HCTR. Baseline characteristics were registered. EuroQol-5D (EQ-5D) and International Physical Activity Questionnaire (IPAQ) were applied at three times: T0 – phase 2 onset; T1 – phase 3 onset; T2 – 3 months after T1.

**Results:**

59 patients participated (Group 1 – 27; Group 2 – 32). We found significant between-group differences regarding occupation (p=0.003). Diabetic patients were less likely to self-select into HCTR (OR=0.21; p<0.05). EQ-5D visual analogue scale and IPAQ result significantly improved between T0-T2 only for HCTR (p=0.001; p=0.021).

**Conclusions:**

HCTR was superior to CCRP on physical activity indices and QoL of ACS patients.

Cardiac rehabilitation (CR) is a multidisciplinary intervention for acute coronary syndrome (ACS) secondary prevention, embracing different core components: patient education, health behaviour modification, exercise training, psychosocial management and vocational support (Scherrenberg et al., 2020; Thomas et al., 2019). CR typically consists of three phases: phase 1 (in-hospital), phase 2 (core components delivery as an inpatient, outpatient or home-based service) and phase 3 (maintenance phase, with the aim of sustaining lifestyle changes) (Scherrenberg et al., 2020). Being a class IA recommendation, CR reduces cardiovascular mortality and hospital admissions, improving quality of life (Anderson et al., 2016; Thomas et al., 2019). Despite these benefits, participation rates remain suboptimal and many patients carry on with a sedentary lifestyle and harmful habits (Kotseva et al., 2019; Santiago de Araújo Pio et al., 2019). One obstacle to CR adherence is a lack of patient self-confidence for lifestyle change (De Bacquer et al., 2022). It has been proposed that the use of mobile applications can be helpful (Burke et al., 2015). Hybrid cardiac telerehabilitation (HCTR) has recently emerged as an innovative model, including both centre-based and home-based interventions (Thomas et al., 2019).

Currently, the recurrent rate of ACS events remains high, due to low CR program (CRP) uptake and reduced long-term adoption of secondary prevention measures (Scherrenberg et al., 2020). Besides, the COVID-19 pandemic is considered a major CRP participation barrier (Scherrenberg et al., 2020). Home-based CR has been proposed as a solution for overcoming these barriers and is well established in the literature as an equally effective alternative to centre-based CR (Anderson et al., 2017).

However, there are few high-quality studies evaluating hybrid models of CR (i.e., centre-based plus home-based interventions). In theory, these offer the best of both worlds in a compelling format (Thomas et al., 2019). Besides, HCTR evidence is poor in southern-European countries (Scherrenberg et al., 2020). In fact, remote interventions seem to have a greater effect on patient adherence, while in-person services may have a greater impact on patient enrollment (Santiago de Araújo Pio et al., 2019). A HCTR trial (Frederix et al., 2015) demonstrated that standard CR plus telerehabilitation leads to a greater improvement in physical fitness and QoL. Apart from that, data regarding predictors of patient self-selection between hybrid and conventional programs is lacking in the literature.

This study had two aims. First, to evaluate the effectiveness of a HCTR program on quality of life (QoL) and physical activity indices among ACS patients throughout phases 2 and 3, and second, to establish predictors for hybrid program self-selection.

## Methods

A single-centre longitudinal unblinded retrospective study included patients who attended a cardiac rehabilitation program (CRP) after ACS at Leiria Hospital Centre between August 2018 and July 2021.

Patients self-selected between two groups. Group 1 attended conventional CRP (CCRP) and Group 2 engaged in HCTR. CCRP consisted of a 12-week phase 2 program, including monitored physical exercise hospital sessions twice a week, as well as weekly relaxation and educational sessions delivered at the hospital. After completing phase 2, Group 1 was clinically advised to maintain lifestyle recommendations throughout phase 3. Group 2 attended HCTR, which included the same CCRP associated to a tele-monitorization platform consisting of a free mobile application, MOVIDA.eros (Silva et al., 2018), that involves physical exercise medical prescription and remote interaction with patients. Tele-monitorization began on phase 2 and participants were asked to register every exercise session on the App. Patients maintained MOVIDA.eros use and were advised to maintain lifestyle recommendations throughout phase 3.

Evaluations were performed three times for both groups: T0 – phase 2 onset; T1 – end of phase 2/phase 3 onset; and T2 – 3 months after phase 3 onset. Baseline characteristics were registered at T0, including age, gender, occupation (intellectual and scientific professionals, administrative staff and the like, services and sellers, agriculture and fishing, workers and craftsmen, retired, unemployed), cardiovascular risk factors (smoking, hyperlipidaemia, hypertension, diabetes mellitus) and Hospital Anxiety and Depression Scale [HADS, Anxiety (A) and Depression (D) scores – normal: 0-7; suggestive: 8-10; probable: 11-21]. Body mass index (BMI) and application of EuroQol-5D (EQ-5D) questionnaire (with registration of EQ-5D index score validated for Portuguese population and EQ-5D visual analogue scale result) and International Physical Activity Questionnaire – Short Form (IPAQ, result expressed in MET-minutes per week) were performed at three times. Estimated functional capacity (EFC) in cardiac stress test was recorded at T0 and T1. Patients who did not attend CRP phases 2 or 3 were excluded. All patients provided informed verbal consent.

Statistical analysis was performed through RStudio 1.3.2056. Descriptive statistics were performed for both groups. Fisher's test and two sample t-test were used to investigate between-group differences for categorical and continuous variables, respectively. Repeated measures ANOVA was applied for comparisons between times for each group, including Friedman and Durbin-Conover test (for pairwise comparisons). Logistic regression was also performed in order establish a probability model regarding variables with statistically significative difference between groups. All p-values inferior to 0.05 were considered statistically significant.

## Results

We enrolled 74 patients and analysed the data of 59 patients who completed CRP phases 2 and 3: 27 on Group 1 and 32 on Group 2 (Table 1).

**Table 1.** Baseline Characteristics of Patients

	Group 1: CCRP (n=27)	Group 2: HCTR (n=32)	p
**Age, years**	59±9	52±9	0.003
**Gender**			1
**Female**	15% (4)	16% (5)	
**Male**	85% (23)	84% (27)	
**Smoking**			0.843
**Current smoker**	30% (8)	34% (11)	
**Prior smoker**	41% (11)	44% (14)	
**Non-smoker**	30% (8)	22% (7)	
**Hyperlipidaemia**	81% (22)	81% (26)	1
**Hypertension**	74% (20)	63% (20)	0.409
**Diabetes mellitus**	33% (9)	9% (3)	0.048
**Family history**	26% (7)	41% (13)	0.774
**BMI, kg/m^2^**	28±6	28±4	0.561
**HADS – A**			0.377
**Normal (0-7)**	57% (13)	76% (22)	
**Suggestive (8-10)**	30% (7)	17% (5)	
**Probable (11-21)**	13% (3)	7% (2)	
**HADS – D**			0.511
**Normal (0-7)**	83% (19)	83% (24)	
**Suggestive (8-10)**	17% (4)	10% (3)	
**Probable (11-21)**	0% (0)	7% (2)	
**Occupation**			0.003
**Intellectual and scientific professionals**	0% (0)	19% (6)	
**Administrative staff and the like**	0% (0)	3% (1)	
**Services and sellers**	22% (6)	42% (13)	
**Agriculture and fishing**	4% (1)	0% (0)	
**Workers and craftsmen**	22% (6)	23% (7)	
Retired	44% (12)	13% (4)	
**Unemployed**	7% (2)	0% (0)	

*Note*. Continuous variables are expressed as mean ± standard deviation. Categorical variables are expressed as proportion in % (n). CCRP: Conventional Cardiac Rehabilitation Program; HCTR: Hybrid Cardiac Telerehabilitation; BMI: Body Mass Index; HADS: Hospital Anxiety and Depression Scale; A: Anxiety; D: Depression.

Continuous variable analysis revealed similar T0 characteristics between groups except for age (p=0.003; CCRP: 59±9 vs HCTR: 52±9). This variable did not correlate with EQ-5D or IPAQ results. Logistic regression model also revealed statistically significant difference in age (p=0.006), as younger patients were more likely to integrate HCTR and each year increase resulted in a 9% lower HCTR self-selection probability. Besides, for patients older than 58, this probability inverted (those were more likely to undergo CCRP).

We verified significant differences in occupational category between Group 1 and 2 (p=0.003). There was a greater proportion of intellectual professionals and a smaller proportion of patients who were retired in Group 2. Diabetic patients were less likely to self-select into HCTR (odds ratio [OR]=0.21; p<0.05).

**Figure 1:**
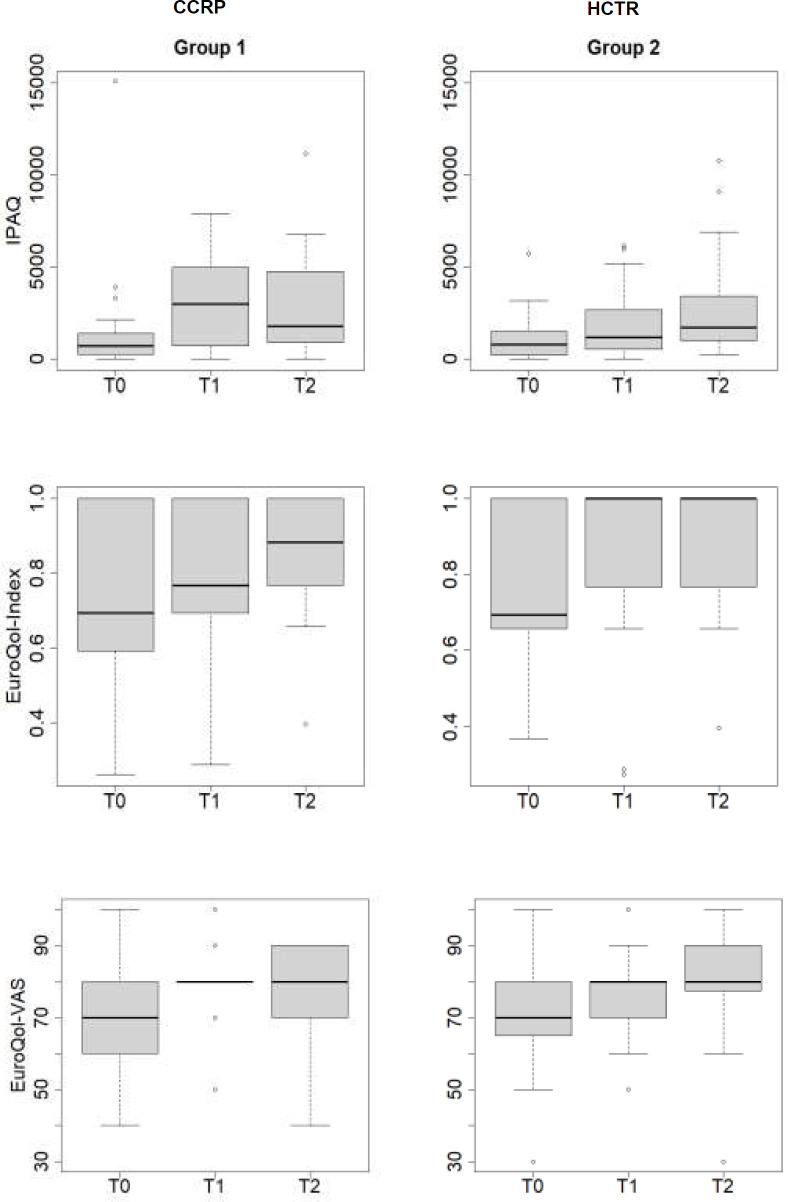
Boxplots Representing IPAQ Result, EQ-5D Index and EQ-5D VAS Evolution throughout Three Times for Both Groups

*Note*. CCRP: Conventional Cardiac Rehabilitation Program; HCTR: Hybrid Cardiac Telerehabilitation.; IPAQ: International Physical Activity Questionnaire – Short Form (result expressed in MET-minutes per week); EQ-5D: EuroQol questionnaire; VAS: Visual analogue scale.

Both groups showed significant EQ-5D index improvements between T0-T1 and T0-T2 (CCRP: p=0.006; HCTR: p=0.002). The EQ-5D visual analogue scale (VAS) result significantly improved between T0-T2 in HCTR (p=0.001 vs CCRP: p=0.126). There was a significant IPAQ result improvement between T0-T2 for HCTR (p=0.021 vs CCRP: p=0.060). These results are illustrated in Figure 1. There were no significant differences on BMI results. EFC improved between T0 and T1 in both groups (p<0.05).

## Discussion

Although both interventions were beneficial, HCTR was superior to CCRP on QoL of ACS patients throughout phases 2 and 3. Only HCTR improved EQ-5D VAS between T0-T2 and both interventions improved EQ-5D index. However, by analysing Figure 1, we see that EQ-5D index improves more rapidly and sustainably for HCTR. HCTR was also superior to CCRP on physical activity indices along both phases. These data favour inclusion of a mobile platform allowing supervision and patient-clinician interaction, so enhancing physical exercise adherence. These results corroborate the previous cited HCTR trial (Frederix et al., 2015). Another advantage of this platform, from the clinician's perspective, is to provide an asynchronous telerehabilitation model, enhancing patient engagement and outcomes.

Study limitations include a possible bias, since the Group 2 population was younger, healthier, and less likely to be retired, all of which likely contributed to their better outcomes. However, this group was also superior on physical activity indices, which means that despite having less free time (inferior proportion of retired) they still performed better on this outcome. This fact highlights the importance of MOVIDA.eros. Our study results suggest that younger adults, with lower incidence of diabetes, who work in intellectual and scientific careers, are more likely to self-select into the hybrid program. These findings add new insights to the existing literature regarding HCTR. Also, this study reinforces hybrid programs evidence by analysing patients from a southern-European country, where HCTR literature is poor (Scherrenberg et al., 2020). Digital literacy (including the use of smartphones with apps) is becoming a decisive factor on good health practices, including cardiovascular disease secondary prevention. Future studies evaluating HCTR are needed, especially on diabetic patients and regarding different occupations.

## Conclusions

HCTR may be beneficial, mostly by improving access to healthcare. Younger adults, with lower incidence of diabetes, who work in intellectual and scientific careers, are more likely to self-select into hybrid programs.
